# Two new species of *Poeciloneta* Kulczyński, 1894 (Araneae, Linyphiidae) from Xizang, China

**DOI:** 10.3897/zookeys.1296.205589

**Published:** 2026-10-28

**Authors:** Yong-Qiang Xu, Muhammad Irfan, Zhi-Sheng Zhang

**Affiliations:** 1 Institute of Plateau Biology of Xizang Autonomous Region, Lhasa 850001, Xizang Autonomous Region, China Institute of Plateau Biology of Xizang Autonomous Region Lhasa China; 2 Medog Biodiversity Observation and Research Station of Xizang Autonomous Region, Medog, China Medog Biodiversity Observation and Research Station of Xizang Autonomous Region Medog China; 3 Key Laboratory of Eco-environments in Three Gorges Reservoir Region (Ministry of Education), School of Life Sciences, Southwest University, Chongqing 400715, China Key Laboratory of Eco-environments in Three Gorges Reservoir Region (Ministry of Education), School of Life Sciences, Southwest University Chongqing China; 4 College of Plant Protection, Southwest University, Chongqing 400715, China College of Plant Protection, Southwest University Chongqing China

**Keywords:** Description, Lepthyphantinae, morphology, sheet-web spiders, taxonomy

## Abstract

Two new species of the genus *Poeciloneta* Kulczyński, 1894 are described from Xizang Autonomous Region: *P. duinanensis***sp. nov**. (♂♀) and *P. jidingensis***sp. nov**. (♂♀). Detailed descriptions, along with photographs of genital characters and somatic features, and a distribution map, are provided. These discoveries increase the known diversity of *Poeciloneta* in China and indicate that linyphiid diversity on the Tibetan Plateau remains incompletely documented. Both species were collected from high-elevation habitats (approximately 3910–4492 m), highlighting the need for continued taxonomic exploration of extreme environments in the eastern Himalayas. This study contributes to understanding the diversity, distribution, and morphological variation of *Poeciloneta* and emphasizes the importance of further integrative taxonomic research in this biologically diverse but poorly sampled region.

## Introduction

The genus *Poeciloneta* comprises 14 species with a predominantly Holarctic distribution, occurring widely across the Nearctic and Palaearctic regions (WSC 2026). In the Nearctic, the genus is represented in Canada and the United States (including Alaska), with *P. variegata* (Blackwall, 1841) also having a broader distribution beyond the region (WSC 2026). In the Palaearctic, *Poeciloneta* is widely distributed from Europe and European Russia eastward through Middle Siberia to the Russian Far East, and southward to Georgia, Iran, Kazakhstan, and Kyrgyzstan, comprising numerous species across this broad region (WSC 2026). In East Asia, *Poeciloneta* is represented by three species in China: *P. variegata* distributed in northern and western China (Qinghai, Xinjiang, and Xizang provinces), and two species described from the Tibetan Plateau, *P. xizangensis* Zhai & Zhu, 2008, and *P. ancora* Zhai & Zhu, 2008, both known from high-elevation habitats around Lhasa, Xizang, at approximately 3655 m (Zhai and Zhu 2008).

*Poeciloneta* species occupy diverse habitats from lowland vegetation to high-elevation mountains, occurring under stones, among mosses and grasses, and in leaf litter in moist habitats and spruce forests (Roberts 1995). The wide ecological and elevational range highlights the considerable adaptability of the genus across its extensive Holarctic distribution. The known distribution of the genus in China is further expanded by two new species described here from Yadong and Sajia counties in Xizang Autonomous Region, highlighting the potential for undiscovered diversity in the region.

## Material and methods

The specimens were collected by sieving leaf litter. All specimens were preserved in 75% ethanol. Left male pedipalps were examined and photographed after dissection. Following dissection, the epigynes were cleared in a trypsin solution before examination and photography. The specimens were examined and measured using a Leica M205A stereomicroscope equipped with a Leica DFC450 camera and LAS v. 4.6 software. All the photos of habitus and genitalia were taken with a Kuy Nice CCD camera mounted on an Olympus BX53 compound microscope. Compound focus images were generated using Helicon Focus v. 6.7.1. Eye sizes were measured at the maximum dorsal diameter. Leg measurements are shown as total length (femur, patella, tibia, metatarsus, tarsus). All measurements are given in millimeters. Specimens are deposited in the School of Life Sciences, Southwest University, Chongqing (**SWUC**), China. The morphological terminology follows Saaristo and Tanasevitch (1996).

The following abbreviations are used in the text and figures: **a.s.l.** = above sea level; **AER** = anterior eye row; **ALE** = anterior lateral eyes; **AME** = anterior median eyes; **AME–ALE** = the distance between AME and ALE; **AME–AME** = the distance between AMEs; **CO** = copulatory opening; **E** = embolus; **EP** = embolus proper; *sensu*Kulczyński (1898); **LC** = lamella characteristica; **LC1** = LC branch 1; **LC2** = LC branch 2; **LL** = lateral lobe; **LP** = lateral pocket; **MM** = median membrane *sensu* van Helsdingen (1965); **PC** = paracymbium; **PCA** = proximal cymbial apophysis; PH = pit-hook *sensu*Saaristo (1971): **PER** = posterior eye row; **PLE** = posterior lateral eyes; **PME** = posterior median eyes; **PME–PLE** = distance between PME and PLE; **PME–PME** = distance between PME; **PMP** = posterior median plate *sensu* van Helsdingen et al. (1977); **PS** = proscapus; **R** = radix; **S** = spermatheca; **ST** = subtegulum; **St** = stretcher; **T** = tegulum; **TA** = terminal apophysis; **TH** = thumb; TmI = position of trichobothrium on metatarsus I.

## Taxonomy

### Family Linyphiidae Blackwall, 1859

**Subfamily Lepthyphantinae Simon, 1929**

#### Genus Poeciloneta

Taxon classificationAnimaliaAraneaeLinyphiidae

Kulczyński, 1894

9FE7209C-431C-55F2-AA1E-96A099612430

##### Name in Chinese.

杂色皿蛛属.

##### Type species.

*Neriene variegata* Blackwall, 1841; gender feminine.

##### Diagnosis.

*Poeciloneta* resembles *Lepthyphantes* Menge, 1866, but differs by a very long, narrow lamella characteristica that splits into several parallel, spine-shaped branches. *Poeciloneta* females are distinguished by their proscape and spinulated metatarsi (Zhai and Zhu 2008).

#### Poeciloneta duinanensis sp. nov.

Taxon classificationAnimaliaAraneaeLinyphiidae

A28DC155-4B38-539E-9663-EA36F4E9E799

https://zoobank.org/71E4D6D1-161F-4F19-A30B-29FAC0F50DE8

**Figure 1.**
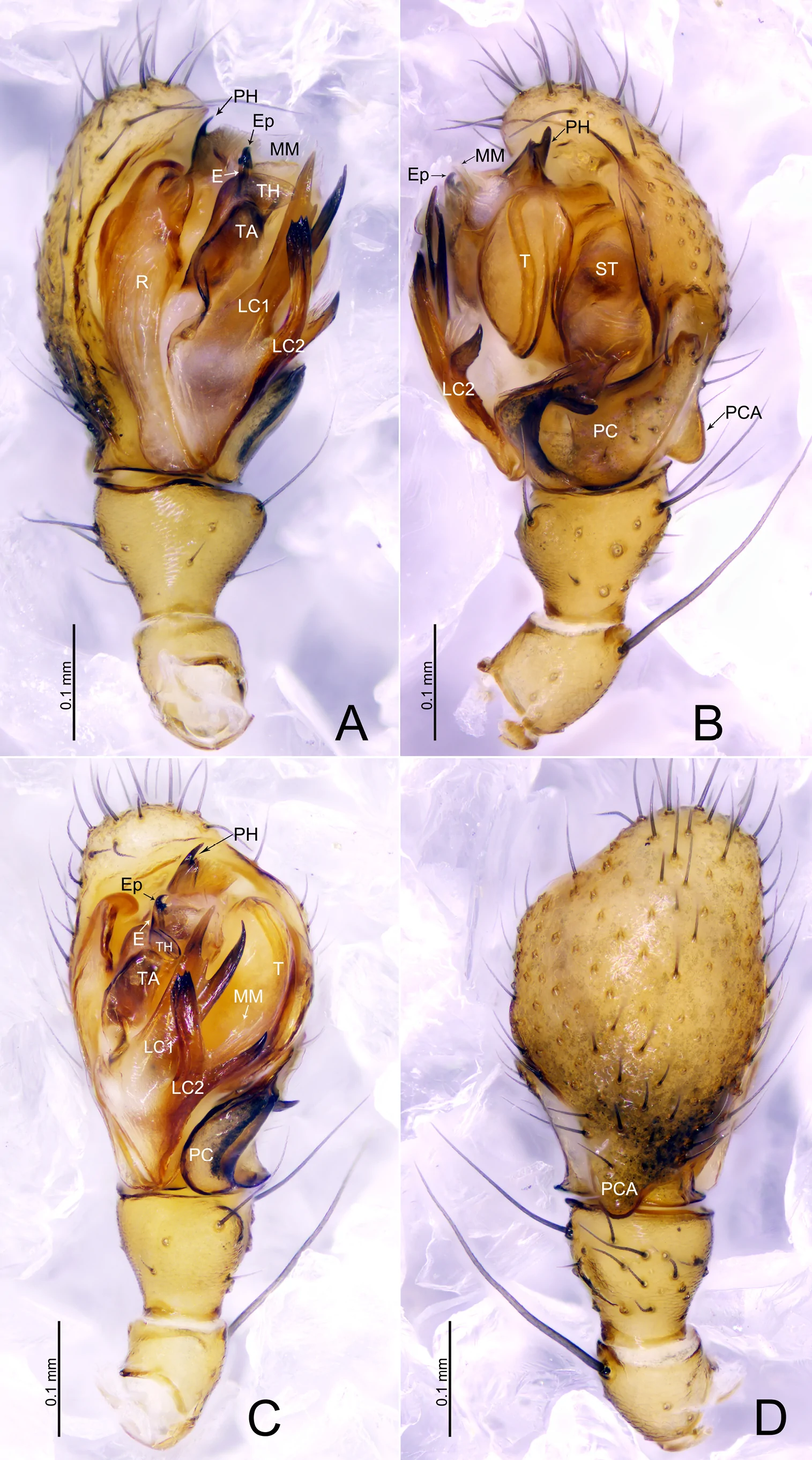
*Poeciloneta duinanensis* sp. nov., male holotype, pedipalp. **A**. Prolateral view; **B**. Retrolateral view; **C**. Ventral view; **D**. Dorsal view.

**Figure 2.**
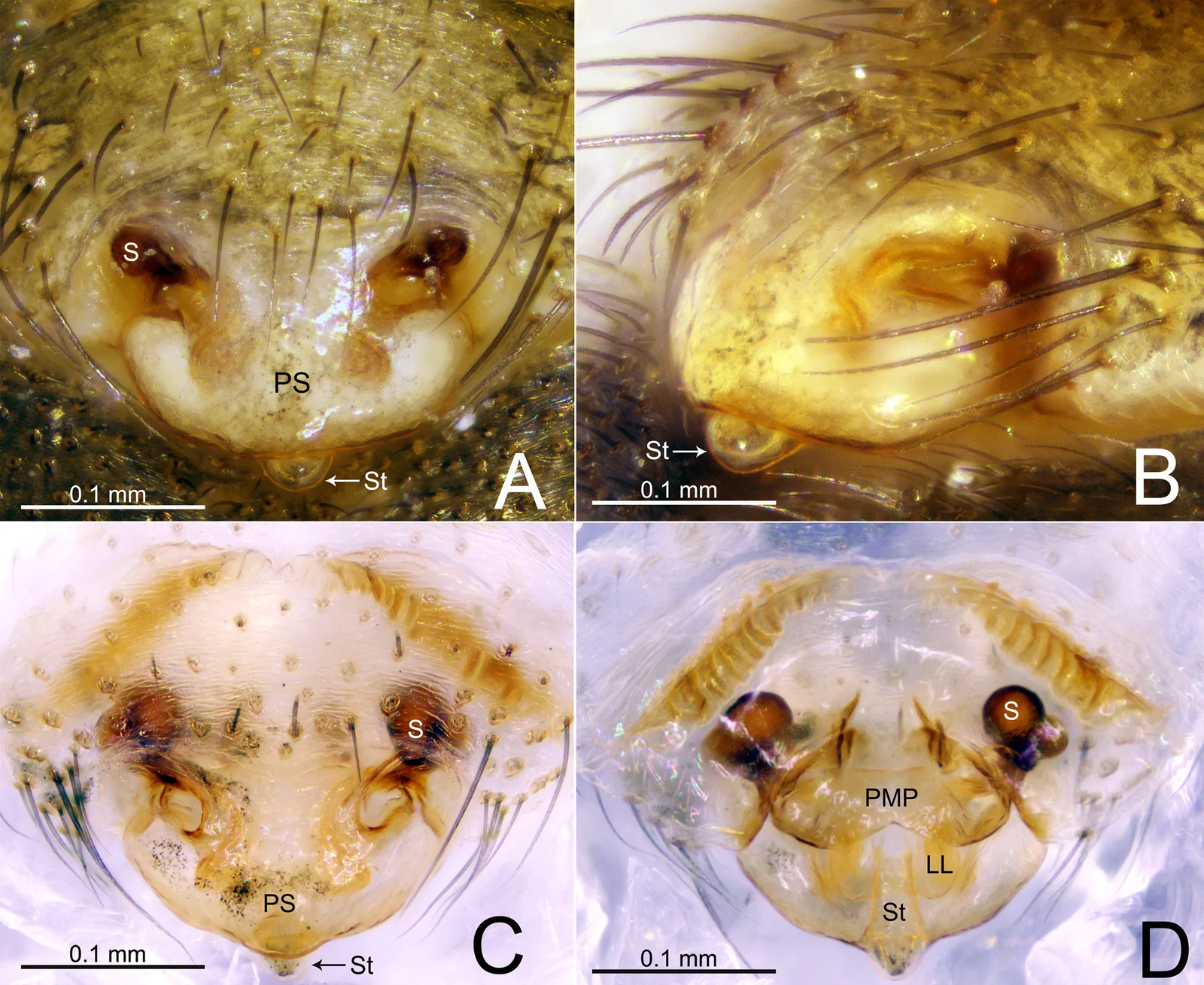
*Poeciloneta duinanensis* sp. nov., female paratype, epigyne. **A**. Ventral view; **B**. lateral view; **C**. Ventral view; **D**. Dorsal view.

Figs 1, 2, 5 A, 6A–D, 7

##### Name in Chinese.

堆纳杂色皿蛛.

##### Type material.

***Holotype*** • ♂; China, Xizang Autonomous Region, Yadong County, Duinan Township (grassland and walls along water’s edge), 27°58.555'N, 89°14.576'E, 4492 m a.s.l., 10 Jul. 2018, Luyu Wang, Zhisun Wu, and Yannan Mu leg. (SWUC-T-LIN-38-01). ***Paratypes***: • 1♂13♀, same distribution data as holotype (SWUC-T-LIN-38-02 to 15).

##### Etymology.

This epithet derives from the type locality.

##### Diagnosis.

**Figure 3.**
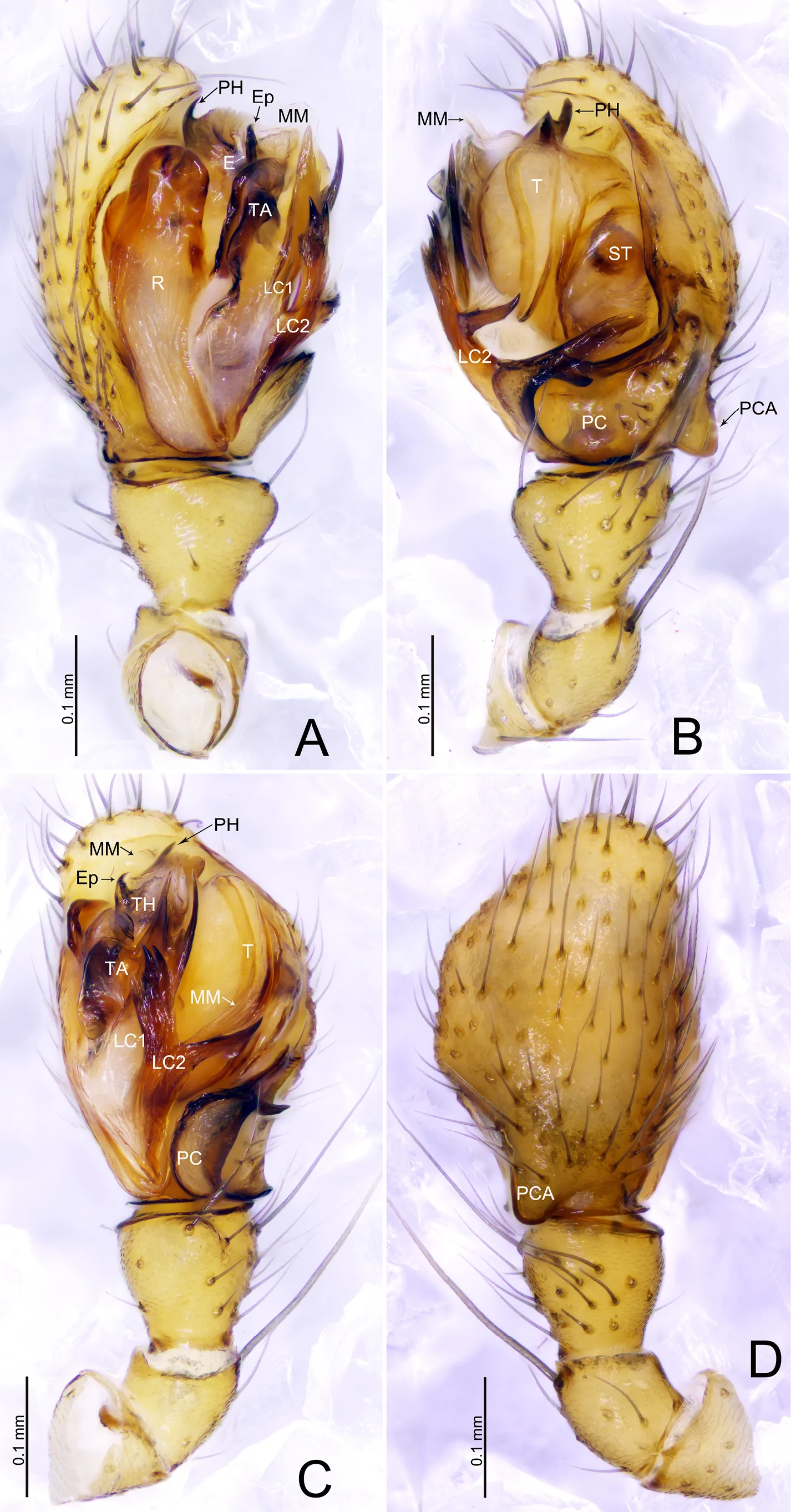
*Poeciloneta jidingensis* sp. nov., male holotype, pedipalp. **A**. Prolateral view; **B**. Retrolateral view; **C**. Ventral view; **D**. Dorsal view.

**Figure 4.**
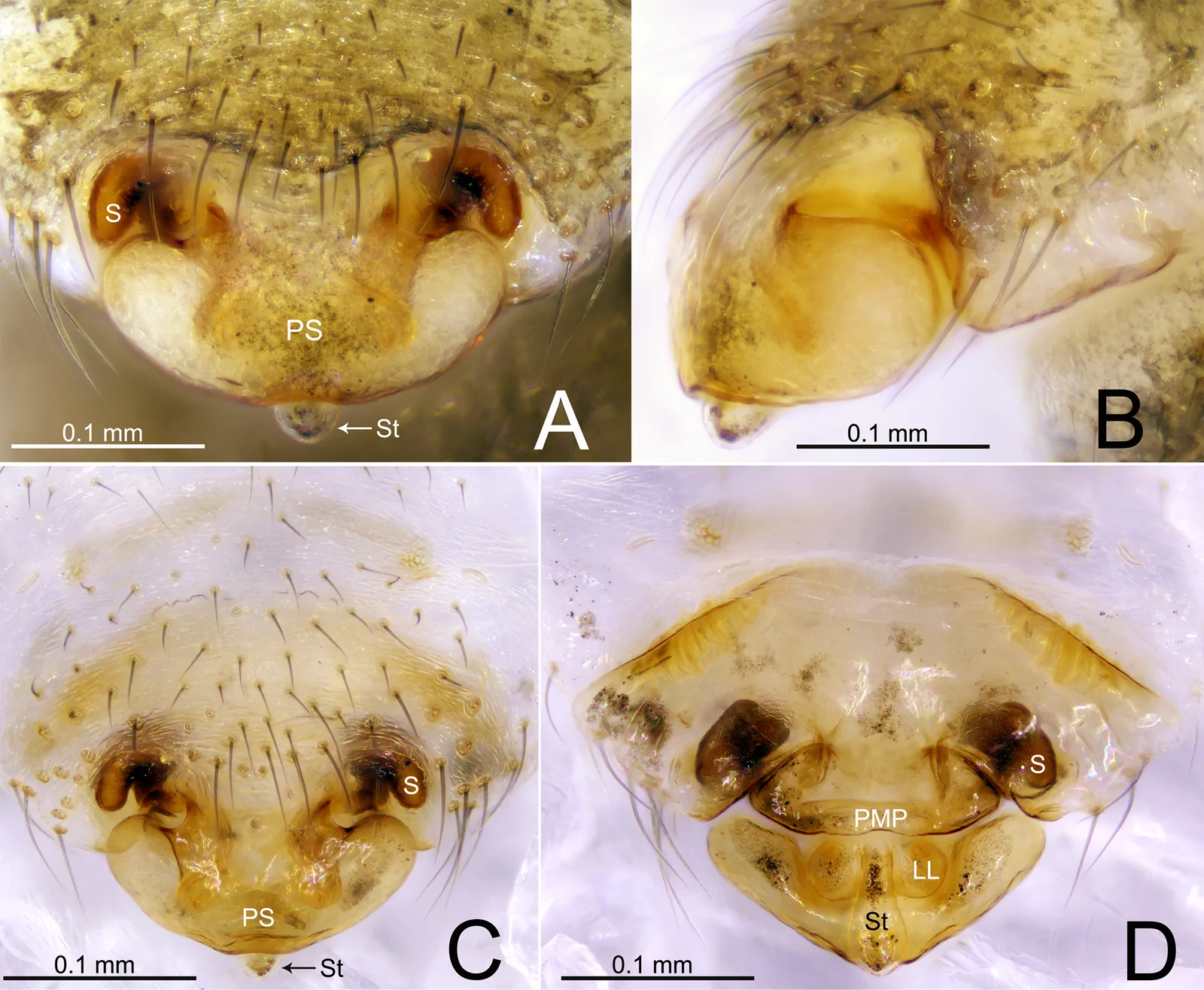
*Poeciloneta jidingensis* sp. nov., female paratype, epigyne. **A**. Ventral view; **B**. Lateral view; **C**. Ventral view; **D**. Dorsal view.

**Figure 5.**
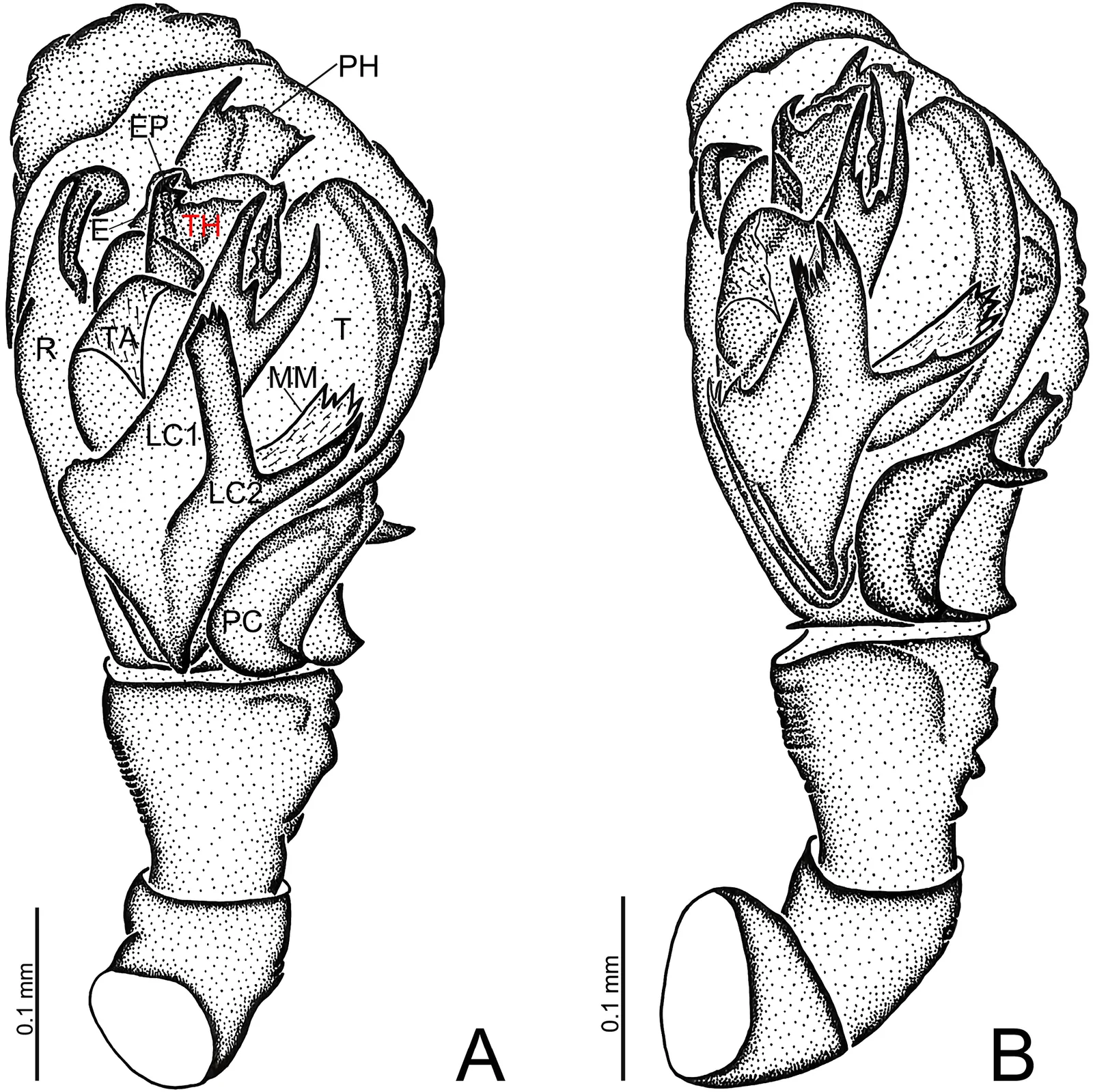
Holotype males of new species of *Poeciloneta*, ventral view. **A**. *P. duinanensis* sp. nov.; **B**. *P. jidingensis* sp. nov.

**Figure 6.**
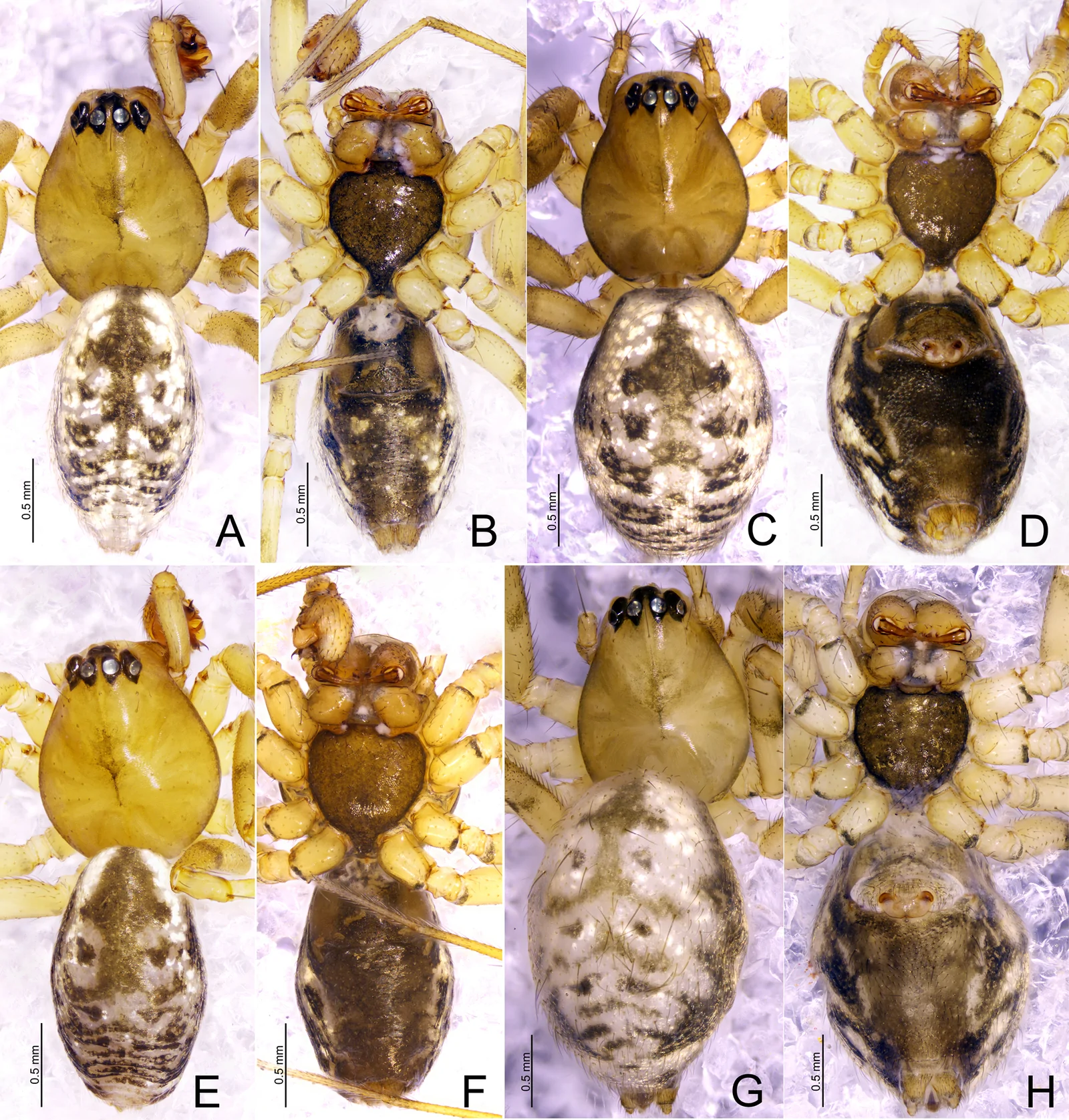
Habitus of new species of *Poeciloneta*. **A**. *P. duinanensis* sp. nov., male holotype, dorsal view; **B**. Ibid., ventral view; **C**. *P. duinanensis* sp. nov., female paratype, dorsal view; **D**. Ibid., female paratype, ventral view. **E**. *P. jidingensis* sp. nov., male holotype, dorsal view; **F**. Ibid., ventral view; **G**. *P. duinanensis* sp. nov., female paratype, dorsal view; **H**. Ibid., female paratype, ventral view.

The male of *Poeciloneta duinanensis* sp. nov. resembles *P. ancora*, *P. jidingensis* sp. nov., and *P. xizangensis* in having a similar distal arm of the paracymbium (PC) and lamella characteristica (LC) with several branches (Figs 1A–D, 3A–D, 5A, 5B; Zhai and Zhu 2008: figs 5–7, 12–14), but it can be distinguished by the LC2 tip, which has three teeth (vs seven teeth in *P. jidingensis* sp. nov. or bifurcated in both *P. ancora* and *P. xizangensis*; compare Fig. 1C with Fig. 3C, Zhai and Zhu 2008: figs 6, 13); LC2 lateral projection tooth absent (vs present in *P. jidingensis* sp. nov.; Figs 1A–D, 3A–D, 5A, 5B)); tip of embolus proper (EP) bifurcated (vs not bifurcated; compare Fig. 1C with Figs 3C, 5A, 5B; figs 6, 13). The female of *P. duinanensis* sp. nov. resembles *P. jidingensis* sp. nov. and *P. xizangensis* in having a similar proscape (PS) (Figs 2A–D, 4A–D; Zhai and Zhu 2008: figs 2–4), but it can be distinguished by the posterior median plate (PMP), which is strongly depressed at the center and has the lateral margin rounded (vs trapezoid in *P. jidingensis* sp. nov. or slightly depressed at center and lateral margin angular in *P. xizangensis*; compare Fig. 2D with Fig. 4D, Zhai and Zhu 2008: fig. 3).

##### Description.

**Male** (holotype, Fig. 6A, B): total length 2.43; carapace 1.19 long, 0.87 wide, yellowish brown; cephalic region slightly elevated; cervical and radial grooves distinct. Clypeus 0.19 high. Chelicerae with two promarginal and three retromarginal teeth. Sternum blackish brown, longer than wide, with microsetae. Eyes: AER recurved, PER straight, slightly wider than AER. Eye sizes and interdistances: AME 0.06, ALE 0.09, PME 0.09, PLE 0.09, AME–AME 0.03, PME–PME 0.05, AME–ALE 0.06, PME–PLE 0.06, AME–PME 0.09, ALE–ALE 0.40, PLE–PLE 0.42, ALE–PLE contiguous. Length of legs: I 5.14 (1.26, 0.34, 1.30, 1.35, 0.89), II 4.69 (1.21, 0.32, 1.21, 1.18, 0.77), III 3.59 (1.01, 0.27, 0.86, 0.89, 0.56), IV 4.75 (1.28, 0.31, 1.23, 1.22, 0.71). TmI 0.26. Tibial spine formula: 2-2-2-2. Opisthosoma 1.38 long, 0.80 wide, oval; dorsal side with greenish pattern extending laterally; lateral side with white patches; ventral side blackish brown with white patches.

***Pedipalp*** (Figs 1A–D, 5A). Patella two-thirds length of tibia, dorsally with a long, thick spine. Tibia conical, with two retrolateral and one dorsal trichobothria. Proximal cymbial apophysis (PCA) triangular, with blunt end. Paracymbium (PC) sclerotized, U-shaped; distal arm tip broadened with blunt end. Pit-hook (PH) relatively strong, sclerotized; distal end grooved. Lamella characteristica (LC) with two branches: LC1 extending forward, with two long branches and an apical median tooth; LC2 tip sclerotized, with three apical teeth; LC2 lateral branch short, with a pointed end. Terminal apophysis (TA) distal end wavy, slightly curved. Embolus (E) long; embolus proper (EP) with bifurcated tip. Thumb (TH) sheet-like.

**Female** (paratype, SWUC-T-LIN-38-02, Fig. 6C, D): total length 3.26; carapace 1.54 long, 1.27 wide, cephalic region slightly elevated, brown, fovea, cervical and radial grooves distinct. Clypeus 0.25 high. Chelicerae with five promarginal and four retromarginal teeth. Sternum longer than wide, dark brown, with microsetae. Eyes: AER recurved, PER straight, slightly wider than AER. Eye sizes and interdistances: AME 0.07, ALE 0.12, PME 0.09, PLE 0.10, AME–AME 0.03, PME–PME 0.04, AME–ALE, 0.07, PME–PLE 0.09, AME–PME 0.11, ALE–ALE 0.47, PLE–PLE 0.49, ALE–PLE contiguous. Length of legs: I 6.10 (1.66, 0.46, 1.58, 1.46, 0.94), II 5.43 (1.53, 0.34, 1.39, 1.31, 0.86), III 4.33 (1.26, 0.36, 1.02, 1.10, 0.59), IV 5.75 (1.69, 0.41, 1.48, 1.41, 0.76). TmI 0.23. Tibial spine formula: 2-2-2-2. Opisthosoma 2.17 long, 1.33 wide, oval, all other characteristics same as in male.

***Epigyne*** (Fig. 2A–D). Proscapus (PS) 1.5 wider than long; stretcher (St) longer than wide in dorsal view, with rounded end; lateral lobe (LL) ear-shaped. Posterior median plate (PMP) heart-shaped, with a round lateral margin. Copulatory opening (CO) present in lateral lobes at the middle of proscapus (PS), anteriorly. Spermathecae (S) tubular, slightly curved, pointing downward.

##### Distribution.

**Figure 7.**
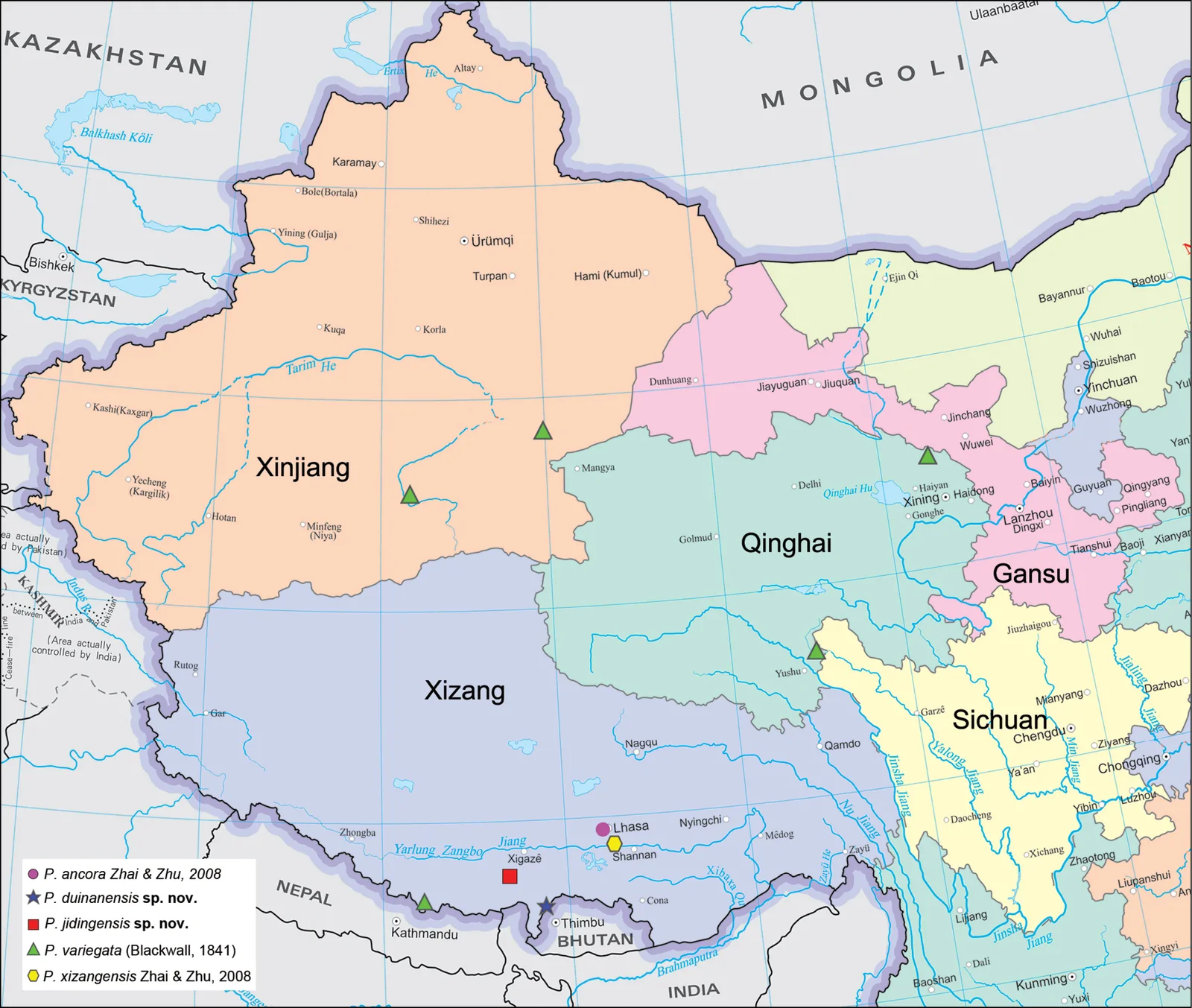
Distribution of *Poeciloneta* species in China.

Known only from the type locality (Fig. 7).

#### Poeciloneta jidingensis sp. nov.

Taxon classificationAnimaliaAraneaeLinyphiidae

7990DCF5-3E6B-55AF-9355-A9F002DB0F50

https://zoobank.org/2CD959D6-2DDA-450C-A2C4-AB8B06302CB1

Figs 3, 4, 5 B, 6E–H, 7

##### Name in Chinese.

吉定杂色皿蛛.

##### Type material.

***Holotype*** • ♂; China, Xizang Autonomous Region, Sajia County, Jiding Town (grassland along farmland), 29°12.571'N, 88°22.267'E, 3910 m a.s.l., 13 Jul. 2018, Luyu Wang, Zhisun Wu, and Yannan Mu leg. (SWUC-T-LIN-39-01). ***Paratypes***: • 1♂19♀, same distribution data as holotype (SWUC-T-LIN-39-02~21).

##### Etymology.

This epithet derives from the type locality.

##### Diagnosis.

The male of *Poeciloneta jidingensis* sp. nov. resembles *P. ancora*, *P. duinanensis* sp. nov., and *P. xizangensis* in having the distal arm of paracymbium (PC) similar and the lamella characteristica (LC) with several branches (Figs 1A–D, 3A–D; Zhai and Zhu 2008: figs 5–7, 12–14), but it can be distinguished by the LC2 tip having seven teeth (vs with three teeth in *P. duinanensis* sp. nov. or bifurcated in both *P. ancora* and *P. xizangensis*; compare Fig. 3D, Fig. 1C, with Fig. 5A, B, Zhai and Zhu 2008: figs 6, 13); LC2 lateral projection tooth present (vs absent in *P. jidingensis* sp. nov. (Figs 3A–D, 1A–D, 5A, 5B)); embolus proper (EP) tip not bifurcated (vs bifurcated; compare Fig. 3C, Fig. 1C and Fig. 5A, B, figs 6, 13). The female of *P. jidingensis* sp. nov. resembles *P. duinanensis* sp. nov. and *P. xizangensis* in having a similar proscape (PS) (Figs 4A–D, 2A–D; Zhai and Zhu 2008, figs 2–4), but it can be distinguished by the trapezoidal posterior median plate (PMP) (vs strongly depressed at center and lateral margin round in *P. duinanensis* sp. nov. or slightly depressed at center and lateral margin angular in *P. xizangensis*; compare Fig. 4D with Fig. 2D, Zhai and Zhu 2008: fig. 3).

##### Description.

**Male** (holotype, Fig. 6E, F): total length 2.43; carapace 1.28 long, 0.98 wide, yellowish brown; cephalic region slightly elevated; fovea, cervical, and radial grooves distinct. Clypeus 0.13 high. Chelicerae with two promarginal and three retromarginal teeth. Sternum dark brown, as long as wide, with microsetae. Eyes: AER recurved, PER straight, slightly wider than AER. Eye sizes and interdistances: AME 0.06, ALE 0.10, PME 0.09, PLE 0.09, AME–AME 0.02, PME–PME 0.05, AME–ALE 0.05, PME–PLE 0.05, AME–PME 0.09, ALE–ALE 0.39, PLE–PLE 0.41, ALE–PLE contiguous. Length of legs: I 5.09 (1.36, 0.34, 1.35, 1.21, 0.83), II 4.40 (1.27, 0.31, 1.11, 0.99, 0.72), III 3.65 (1.02, 0.27, 0.86, 0.91, 0.59), IV 4.74 (1.30, 0.29, 1.16, 1.26, 0.73). TmI 0.24. Tibial spine formula: 2-2-2-2. Opisthosoma 1.40 long, 0.84 wide, oval; dorsal side with greenish pattern extending laterally; lateral side with white patches; ventral side blackish brown.

***Pedipalp*** (Fig. 3A–D, 5B). Patella two-thirds of length of tibia, dorsally with a long, thick spine. Tibia conical, with two retrolateral and one dorsal trichobothria. Proximal cymbial apophysis (PCA) triangular, with blunt end. Paracymbium (PC) sclerotized, U-shaped; distal arm tip broadened, with blunt end. Pit-hook (PH) relatively strong, sclerotized; distal end grooved. Lamella characteristica (LC) with two branches: LC1 extending forward, distal branch with two long branches and two median teeth apically; LC2 tip sclerotized, apically with seven teeth and lateral branch short, with a tooth and pointed end. Terminal apophysis (TA) distal end wavy, slightly curved. Embolus (E) long; embolus proper (EP) slightly curved, with pointed tip. Thumb (TH) inconspicuous.

**Female** (paratype, SWUC-T-LIN-39-02, Fig. 6G, H): total length 2.72; carapace 1.25 long, 0.89 wide, cephalic region slightly elevated, yellowish brown, fovea, cervical and radial grooves distinct. Clypeus 0.22 high. Chelicerae with three promarginal and three retromarginal teeth. Sternum longer than wide, blackish brown, with microsetae. Eyes: AER recurved, PER straight, slightly wider than AER. Eye sizes and interdistances: AME 0.07, ALE 0.10, PME 0.08, PLE 0.09, AME–AME 0.02, PME–PME 0.05, AME–ALE, 0.04, PME–PLE 0.05, AME–PME 0.08, ALE–ALE 0.37, PLE–PLE 0.41, ALE–PLE contiguous. Length of legs: I 4.91 (1.32, 0.36, 1.22, 1.18, 0.83), II 4.52 (1.23, 0.35, 1.12, 1.10, 0.72), III 3.50 (0.94, 0.27, 0.83, 0.90, 0.56), IV 4.58 (1.33, 0.29, 1.11, 1.15, 0.70). TmI 0.21. Tibial spine formula: 2-2-2-2. Opisthosoma 2.17 long, 1.33 wide, oval; all other characteristics same as in male.

***Epigyne*** (Fig. 4A–D). Proscapus (PS) 1.5 wider than long; stretcher (St) longer than wide in dorsal view, with rounded end; lateral lobe (LL) globular. Posterior median plate (PMP) trapezoidal. Copulatory opening (CO) present in lateral lobes at the middle of proscapus (PS) anteriorly. Spermathecae (S) tubular, strongly curved.

##### Distribution.

Known only from the type locality (Fig. 7).

## Discussion

Morphologically, *Poeciloneta* shows close affinity with *Lepthyphantes*, but it is distinguished by the unarmed metatarsi each bearing a distal trichobothrium, the absence of tibial lateral spines, a very long, narrow lamella characteristica typically split into several parallel spine-shaped branches, and, in females, a pseudoscape (= proscape) together with spinulated metatarsi (Tanasevitch 1989; Zhai and Zhu 2008). Although the two genera have long been regarded as closely related lineages within a morphologically cohesive Linyphiinae, and Tanasevitch (1989) even suggested treating *Poeciloneta* as a subgenus of *Lepthyphantes*, the diagnostic characters of *Poeciloneta* have remained stable, supporting its status as a distinct genus.

The two new species described here, *P. duinanensis* sp. nov. and *P. jidingensis* sp. nov. from Xizang, fully conform to the generic diagnosis of *Poeciloneta*. Males possess the characteristic multibranched lamella characteristica and females exhibit the typical proscape. By expanding morphological variation within the genus (particularly in the configuration of the lamella characteristica) without altering its core diagnosis, these species reinforce the stability and diagnostic value of these characters at the generic level.

The discovery of *Poeciloneta duinanensis* sp. nov. and *P. jidingensis* sp. nov. increases the number of *Poeciloneta* species recorded from China from three to five and adds to the documented diversity of the genus in Xizang. Collected at elevations of 4,492 m and 3,910 m, respectively, the two species provide additional evidence of the genus’s occurrence in high-elevation habitats on the Tibetan Plateau. However, each species is currently known only from its type locality, leaving its geographic range, elevational limits, and habitat requirements unknown. Further sampling across localities, elevations, and habitat types is needed to assess their distributions and determine whether additional species remain undocumented. Integrating these collections with morphological and molecular analyses would also help clarify species boundaries and evolutionary relationships within the genus.

## Supplementary Material

XML Treatment for Poeciloneta

XML Treatment for Poeciloneta duinanensis

XML Treatment for Poeciloneta jidingensis

## Additional information

### Artificial Intelligence (AI) use

The authors accept full responsibility for the content of the manuscript, including the disclosure of any use of AI.

No AI tools were used in the preparation of this manuscript.

### Author ORCIDs

Y.-Q. Xu https://orcid.org/0000-0002-1889-9531

M. Irfan https://orcid.org/0000-0003-0445-9612

Z.-S. Zhang https://orcid.org/0000-0002-9304-1789

### Data availability

All of the data that support the findings of this study are available in the main text.
